# Development and validation of a driving simulator for evaluating the residual effects of drugs on driving performance – sensitivity analysis using zopiclone as a positive control: Study Protocol Clinical Trial (SPIRIT Compliant)

**DOI:** 10.1097/MD.0000000000019395

**Published:** 2020-03-20

**Authors:** Mari Iwata, Kunihiro Iwamoto, Daiji Kambe, Naoki Tachibana, Masahiko Ando, Norio Ozaki

**Affiliations:** a Department of Psychiatry, Nagoya University, Graduate School of Medicine, Nagoya, Aichi; b Development Planning, Taisho Pharmaceutical Co., Ltd., Tokyo; c Center for Advanced Medicine and Clinical Research, Nagoya University Hospital, Nagoya, Aichi, Japan.

**Keywords:** driving performance, driving simulator, positive control, validation, zopiclone

## Abstract

**Introduction:**

Drugs acting on the central nervous system (CNS), especially hypnotics, can impair driving. The US Food and Drug Administration started requiring pharmaceutical companies to evaluate the residual influence of CNS agents on driving performance to review their recommended doses. Although it is important for physicians to discuss automobile driving while on medication with patients to promote traffic safety, the package inserts of most CNS agents in Japan uniformly prohibit patients from driving. Although more evidence-based information regarding the effects of drugs on driving performance is needed, the current evaluation methods for driving performance abroad cannot be applied directly to Japanese drivers because of differences in traffic environments, laws, and constitutions. Therefore, we plan to establish a new driving simulator (DS) that would enable the next-day residual effects of drugs on driving performance to be examined.

**Methods:**

In this double-blind, randomized, placebo-controlled, crossover trial, we plan to recruit 26 healthy Japanese males aged 21 to 64 years through advertisements. During the test periods, which will take place twice every other week, the participants will undergo a DS evaluation in the hospital for 2 days/1 night after the first and last doses of the study drug following 8 days of administration. The participants in the study drug group will take zopiclone 7.5 mg at bedtime on the first and eighth days in the hospital, and placebo on the other days. The DS evaluation consists of road tracking, car following, and harsh braking tests. The primary outcome is the standard deviation of lateral position (SDLP), which is a gold standard evaluation item, in the 60-min road-tracking test. The exploratory outcomes are other evaluation items in the DS tests, in the Karolinska Sleepiness Scale sleep questionnaire, and the Profile of Mood States Second Edition rating scale. The estimated difference in the SDLP between the zopiclone and placebo groups will then be calculated.

**Trial registration:**

This study was registered at ClinicalTrials.gov NCT 04108351, on September 30, 2019. Ethics approval was obtained from the Ethics Committee at Hakata Clinic and the Nagoya University Medical School Hospital Bioethics Review Committee.

## 1 Introduction

Drugs acting on the central nervous system (CNS) have side effects such as drowsiness, dizziness, light-headedness, and reduced attention, which can impair driving. In regard to CNS agents, epidemiological studies have reported a relationship between traffic accidents and the use of hypnotics, anxiolytics,^[1,2]^ and antidepressants.^[3,4]^ These findings raise serious concerns about the balance between individual rights and public safety when driving under the influence of CNS agents. The World Health Organization has pointed out that the influences of drugs on driving are not negligible, and published a policy brief regarding drug use and road safety.^[5]^ In particular, the effects of hypnotics on automobile driving are attracting increasing attention. The next-day blood concentration of zolpidem, one of the most frequently prescribed hypnotics, may be high enough to impair activities that require attention, such as driving. Therefore, the US Food and Drug Administration (FDA) reduced the recommended dose for zolpidem,^[6,7]^ issued a warning in regard to driving the day after hypnotics use, and reviewed the recommended dose for all hypnotics.^[8]^ As a result, the FDA now requires pharmaceutical companies to evaluate the influences of CNS agents on driving.^[9]^ In regard to the residual effects of hypnotics on driving performance, recently developing hypnotics with antagonistic actions on orexin receptors, suvorexant has been determined its recommended dose.^[10]^ In addition, a CNS agent other than hypnotics, flibanserin was required to study its effects on driving performance and has been approved.^[11]^

In European Union nations, highway driving tests have long been established as an evaluation system for the effects of drugs on driving performance,^[12,13]^ and all drugs are classified into four categories according to degree of risk based on epidemiological and experimental studies and the occurrence of adverse events.^[14]^ Sharing such information in discussions with patients and medical staff is considered important for traffic safety. Evidence-based information regarding drugs and driving is provided in this manner in Europe and the US. However, in Japan, almost all CNS agent package inserts, except those for some antidepressants, uniformly prohibit patients from driving while on medication regardless of the patient's condition and drug-treatment period, and harsher penalties for being involved in traffic accidents while on medication have also been enacted. Due to such uniform regulations, there are few opportunities for medical staff to discuss automobile driving while on medication with patients; thus, the daily lives of many patients in Japan are restricted.

One of the reasons for this lack of opportunities is that no standardized evaluation method for driving performance has been established in Japan. Although many researchers use their own methods for assessing driving performance,^[13]^ no existing method in Japan has been confirmed in terms of validity or reliability. The evaluation system considered the gold standard uses actual vehicles on the highway in the Netherlands.^[12]^ The standard deviation of lateral position (SDLP), which indicates vehicular weaving, has been confirmed in terms of test–retest reliability^[12]^ and validity for alcohol.^[12]^ The validity of this index has also been confirmed for acute, chronic, and next-day residual effects on driving performance after administration of positive control drugs.^[15,16]^ However, this system is only applied on Dutch highways. In addition, the traffic environment in the Netherlands differs from that in Japan; thus, safety and economic issues remain.

A driving simulator (DS) could be expected to resolve these issues. Although a DS evaluation system for the acute effects of alcohol and the residual effects of zopiclone has already been used for new drug applications in the US,^[11]^ this system cannot be applied in Japan because of differences in the traffic environments. In addition, few evaluation systems can assess the next-day residual effects of drugs on driving performance. In other words, the establishment of a DS evaluation system in Japan would enable the effects of drugs on driving performance to be investigated, provide useful information for patients and doctors in the future, and contribute to traffic safety.

With this background, we developed a new DS evaluation system for alcohol and carried out validation, the results of which are currently being analyzed.^[17]^ To confirm the validity of the system,^[18]^ a trial comparing a positive control drug with placebo is required as the next step. Therefore, we planned to establish a new DS evaluation system that assesses the residual effects of drugs on driving performance for Japanese. The primary end point is the SDLP,^[12]^ the validity of which has been repeatedly confirmed. To verify the residual effects of hypnotics, which has become an increasingly popular topic in recent years, this protocol will verify the analytical sensitivity of the evaluation system using zopiclone, which has been used repeatedly as a positive control in actual driving tests.^[19]^ Zopiclone is a commonly used benzodiazepine receptor agonist with well-known residual effects on driving performance, and has been proposed as a positive control for studying the safety of hypnotics.^[12,20–22]^ Zopiclone has been used in previous studies involving DS research to verify the CRCDS Mini-Sim,^[23]^ and as a positive control in actual driving tests to examine the effects of lemborexant,^[24]^ suvorexant,^[10]^ and ramelteon^[25]^ on driving performance. This study was designed to examine, using the new DS, the next-day effects of zopiclone 7.5 mg (taken at bedtime) on driving performance.

## 2 Methods

### 2.1 Study design

This study is planned as a double-blind, randomized, placebo-controlled, crossover trial. Taisho Pharmaceutical Co., Ltd., will be conducting the clinical trial at Fukuoka Mirai Hospital in Japan. The purpose of this study is to evaluate driving performance by repeated administration of hypnotics (zopiclone 7.5 mg), and to confirm that zopiclone has analytical sensitivity as a positive control in the new DS evaluation system. The administration period is 8 days each in test periods ① and ②. A practice period with the same contents as the test periods will be carried out to allow the participants to become accustomed to operating the DS. Each driving performance evaluation will be conducted during a 2-day/1-night hospital stay, with a 7-day interval between tests.

### 2.2 Participants

Healthy Japanese male volunteers will be recruited through advertisements online and at Fukuoka Mirai Hospital. The sample size was set at 26 with reference to previous studies examining the analytical sensitivity of a DS test system.^[20,22,26]^ The inclusion criteria are as follows: age range 21 to 64 (inclusive) years; body mass index 18.5 to 25.0 kg/m^2^; possession of a driver's license and driving on a daily basis for ≥3 years; consistent sleeping pattern (awaken between 06:00 AM and 09:00, go to bed between 09:00 PM and 00:00); no visual impairments; able to operate a DS with a full understanding of all DS tasks; judged by a physician as being able to participate; and able to provide written informed consent before the examination begins. The exclusion criteria are: having a disease recognized as being non-healthy by a physician; a history of drug or food allergies; serious allergic predispositions; a history of hypersensitivity to zopiclone; a history of stroke, head trauma, epilepsy, or malignant tumor; a 3-month or longer history of sleep disorders, a medical history of sleep apnea syndrome or restless legs syndrome, or a history of hypersomnia or narcolepsy; use of any medication, including over-the-counter drugs, within 1 week before starting the practice period; use of sedative hypnotics within 4 weeks before starting the practice period; experiencing a 6-hour or longer time difference from 4 weeks before starting the practice period until test completion; irregular shift work and night shift work within 4 weeks before starting the practice period; experience using the same DS evaluation method as that used in the present study; a daily routine of alcohol consumption until sleep; unable to stop drinking from 1 day before until the day of the screening test, and from 2 days before hospitalization until discharge; smoking during hospitalization; donating blood within 12 weeks before starting the practice period; use of investigational drugs within 4 weeks before starting the practice period; a diagnosis or history of alcoholism or drug dependency; showing a positive drug test result for benzodiazepines, narcotics classified as cocaine or morphine, stimulants, cannabis, barbiturates, phencyclidine, or tricyclic antidepressants; unable or unwilling to comply with the study protocol; and judged unsuitable for participation by a physician. The discontinuance criteria are: noncompliance with the study protocol; experiencing adverse events that compel a physician or the participant himself/herself to cease participation in the trial; choosing to discontinue the trial of one's own volition; unable to be contacted; sliding off the track or having a large SDLP (e.g., ≥60 cm) during the practice period; and judged unsuitable for participation by a physician.

### 2.3 Randomization and blinding

Participants will be randomly assigned to the zopiclone and placebo groups at a ratio of 1:1 (Table 1). Randomization will be conducted based on a computer-generated random number table. The allocation table conducted by an assignment manager will not be disclosed until all data are fixed. Since the zopiclone tablets might be visibly distinguishable from the placebo tablets, they will be placed in an opaque container and administered in a manner to ensure blinding on the test day. The assignment manager and institution for measuring serum zopiclone concentrations will be disclosed to the investigators.

**Table 1:**
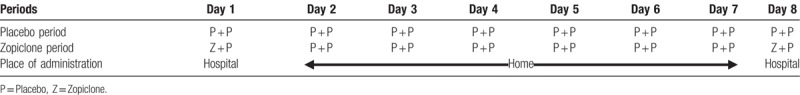
Study drug dosing schedule.

### 2.4 Study drug

Participants will be administered zopiclone 7.5 mg tablets as the active drug and placebo as the control drug. The study drug will be orally administered with 150 mL of water at bedtime on the day before the DS test. The single dose of zopiclone was set to 7.5 mg, which is used in daily medical care, with reference to existing driving tests^[10,23,25]^ and package inserts. Since the tablet shape differs between the active and placebo drugs, it will be necessary to control for participant prejudice (e.g., sight, touch). Therefore, blinding will be maintained by administering both zopiclone and placebo tablets in the zopiclone group and two placebo tablets in the placebo group. For 6 days from the DS evaluation date during the test period, the zopiclone and placebo groups will both be instructed to take two placebo tablets at home. The administration period will be 8 days each in test periods ① and ② (16 days in total). To measure blood exposure, all participants will undergo blood sampling after the DS evaluation.

### 2.5 Test schedule

The test schedule is shown in Table 2. The test is divided into a screening period, a practice period, and two test periods, with 7-day intervals between each period. In each test period, two inspections for 2 days/1 night will be performed on the next day of first and last administration. All participants will be assessed in terms of background characteristics and undergo medical and ophthalmic examinations, a vital sign check, electrocardiogram, blood test, and urinalysis during the screening period. Medical examinations will be performed and vital signs checked at each hospital stay before the DS task and at discharge. The study drug will be administered with 150 mL of water at bedtime on the hospital day, and the serum concentration of zopiclone will be measured the day after the DS evaluation (10.5 hours after administration of the study drug). The participants will also be given the opportunity to become accustomed to operating the DS before each DS task. After awakening, they will complete a sleep questionnaire, the Karolinska Sleepiness Scale (KSS), and the Profile of Mood States Second Edition (POMS 2) before performing the DS task. The timetable for the test period is shown in Table 3.

**Table 2:**
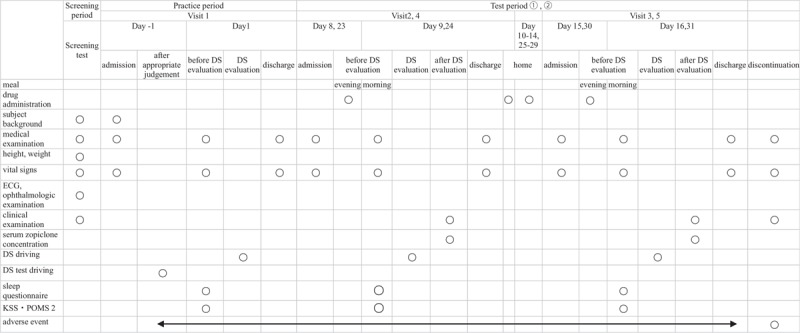
Test schedule.

**Table 3:**
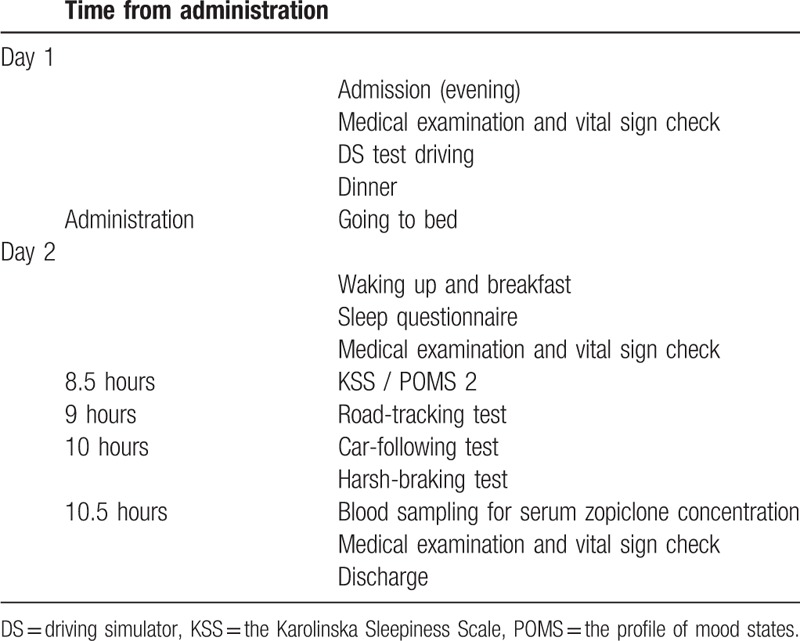
Time schedule of the test period.

### 2.6 DS evaluation

The DS software runs on a Windows PC equipped with a steering wheel, brake pedal, and accelerator system (Driving Force GT; Logicool). The image from the PC is projected onto an 80-in screen using a liquid crystal projector (EB-X05; Epson, Nagano, Japan).

The DS evaluation consists of three tasks. In the first, a road-tracking task, participants are instructed to drive in the center of the left lane while maintaining a speed of 100 km/h on a two-lane road with a gentle curve. The SDLP is the primary measurement. The measurement time is 60 minutes from 9 hours after taking the study drug. In the next task, a car-following test, the participants are instructed to maintain a constant intervehicle distance from a preceding vehicle with varying speeds. When the preceding vehicle decelerates, the brake lights come on. This test measures the distance coefficient of variation (DCV), which is obtained by dividing the standard deviation of the distance between vehicles by the average. The measurement time is 5 minutes from 10 hours after taking the study drug. In the last task, a harsh braking test, the participants are instructed to maintain a constant speed of 50 km/h and to avoid colliding with humanoid models randomly appearing from either side of the road by harsh braking. This test takes about 5 minutes, and is conducted continuously after the completion of the car following task.

### 2.7 Primary outcome

The primary endpoint is the SDLP, which represents the distance from the center line of the road to the right edge of the vehicle body in the road tracking task. This is the most sensitive indicator,^[27]^ and the only one validated and reliable, to evaluate driving ability after the administration of CNS drugs.^[12]^ Since the SDLP has been used as a primary outcome in previous reports to evaluate the effects of drugs on driving performance, it was also set as the primary outcome in the present DS study.

### 2.8 Exploratory outcomes

The following outcomes will be used as exploratory evaluation items: total number of times the car body crosses; the lane (inappropriate line crossing; ILC); total number of times the vehicle goes off of the course (total course-outs; TCO); standard deviation of speed in the road tracking test; the DCV to the preceding vehicle in the car following test; reaction time for detecting deceleration of the preceding vehicle (time to speed adaptation); number of collisions with the preceding vehicle (car collisions) in the car following test; brake reaction time (BRT); and number of collisions with an object (error) in the harsh braking test.

### 2.9 Other outcomes

In addition to the DS evaluation items, an exploratory analysis of the following will also be conducted. The Japanese versions of POMS 2 and the KSS^[28]^ will be performed in consideration of the possibility that drowsiness and mood at the time of the examination could affect the results. The participants will also self-evaluate their sleep (e.g., sleep latency, number of awakenings, wake time after sleep onset, total sleep time) subjectively using questionnaires at the time of awakening.

### 2.10 Statistical analysis

Primarily, basic statistics for 60-minute cumulative SDLP will be calculated, as will basic statistics for the difference (ΔSDLP) between the positive control drug (zopiclone) and placebo groups and the two-sided 90% confidence interval (CI) of the mean difference. If both lower limits of the two-sided 90% CIs of ΔSDLP at each inspection are greater than 0 cm, zopiclone will be judged to have analytical sensitivity. Secondarily, the frequency of ΔSDLP categories will counted and a symmetry analysis will be conducted.

### 2.11 Adverse events

If any adverse events occur after the start of the practice period, the study can be discontinued based on the doctor's or participant's own decision in accordance with severity. Adequate medical care will be provided to the participants in the case of any adverse event. All adverse event data will be monitored and reported in detail at a later date, but will not be aggregated or analyzed.

### 2.12 Ethics and dissemination

This study was registered at ClinicalTrials.gov (NCT 04108351) on September 30, 2019. The study protocol was approved by the Ethics Committee at Hakata Clinic (1747CP-4) and the Nagoya University Medical School Hospital Bioethics Review Committee (2010-970-4). The study will be carried out at Fukuoka Mirai Hospital. Informed consent will be obtained from all study participants. For privacy protection, all participants will be identified using an anonymous identification code. Information such as the participant's name and address will be managed by only the medical examination center, and will not be provided to other organizations. If any necessary experimental data are provided to a joint research institution (investigator and sponsor), these will be carefully protected using only the participants’ identification codes and a corresponding table. The sponsor and investigator will have access to the final test data. The acquisition of informed consent, the inclusion/exclusion criteria, participant eligibility, and the occurrence of any adverse events will be confirmed by monitors from outside the testing agency. These monitors will ascertain whether the experiment is being carried out according to the approved procedure and confirm that the data storage method is appropriate. We will also set up an independent auditor from the testing department to evaluate whether the experiment complies with the protocol. All test-related data will be disclosed to the monitor or auditor for the purposes of conducting a survey. To publicize and explain the research to key audiences, the experimental results will be aimed at publication in a peer-reviewed journal and presented at local, national, and international conferences.

## 3 Discussion

The objective of the proposed study is to verify the analytical sensitivity of a DS evaluation system using zopiclone in healthy Japanese volunteers. Any DS system close to the actual vehicle test in the Netherlands, which is the gold standard for evaluating the effects of drugs on driving performance, must satisfy several conditions, including

1) validation of the acute effects of alcohol drinking,
2) validation of the residual effects of positive control drug administration, and
3) verification of test–retest reliability.

However, no existing DS evaluation system currently satisfies all of these conditions. Although validation with alcohol and positive control drugs are examined in the CRCDS Mini-Sim^[23]^ and DS of Green Dino,^[29]^ it is difficult to apply these DSs to verification in Japan because of differences in driving environments.

Zopiclone is a widely used hypnotic, and its effect on the driving performance has been repeatedly reported.^[19,30,31]^ In fact, an epidemiological study showed that zopiclone users are four times more likely to be involved in traffic accidents.^[32]^ Therefore, in driving studies, zopiclone has been used most frequently as a positive control to validate residual effects.^[13,15]^ In addition, amitriptyline,^[33–35]^ mirtazapine,^[16,36,37]^ and recently, alprazoram,^[29]^ may be used as positive control drugs. We selected zopiclone as the positive control drug in this study because it has been used for not only actual vehicle tests, but also other DS validations.^[23]^ Females are generally more likely than males to be affected by drugs;^[38]^ however, no significant sex or age differences in the effects of zopiclone on driving performance have been reported.^[39]^

The SDLP, as the primary end point, has a threshold equivalent to a blood alcohol concentration (BAC) of 0.05%, which is the legal limit in many countries, and is known to increase in parallel with BAC.^[12]^ In general, the SDLP often shows a larger value in DS systems than in actual vehicle tests. On the other hand, zopiclone 7.5 mg has been reported to increase the SDLP and to have an effect equivalent to a BAC of 0.05 to 0.08%.^[39]^ Most previous studies have found that zopiclone significantly increases the SDLP the day after administration, although the measurement time after administration has varied with respect to each experiment.^[19,22,30,31,40]^ The SDLP measured in the acute phase (5–6 hours after administration) has been reported to be increased,^[41]^ whereas that at 16 hours after administration has not.^[42,43]^ Given this background, and in reference to the previous reports,^[20,22,26]^ the present study will be conducted 9 to 10 hours after administration, at which time driving performance is expected to be affected. In general, hypnotics are known to increase the risk of traffic accidents with increases in drug half-life and to decrease after repeated administration.^[42]^ Zopiclone is an ultra-short-acting hypnotic with a maximum blood concentration time (T_max_) of about 1 hour and a blood elimination half-life (T_1/2_) of about 4 hours. However, since the residual effect of zopiclone on driving performance has been repeatedly reported even at about 9 hours after administration, when the blood elimination half-life is exceeded, it seems to be an appropriate established time for the next day after administration.

Other secondary evaluation items vary based on previous reports, and the results are not necessarily consistent. For example, many studies have reported significant effects of zopiclone on the standard deviation of speed (SDS),^[10,19,21,31,39]^ whereas others have not.^[23,25,30]^ In this study, we included SDS as an exploratory item. Since lane exceedance and road exits, which indicate that the SDLP exceeds a certain level, have had significant effects in some investigations,^[11,21,23]^ this study may reveal increases in ILC and TCO. One prior report found that zopiclone significantly increased the number of collisions in the car following test.^[11]^ As the DCV is considered to predict the number of collisions, it is therefore likely to be a predictor of accident risk; thus, measuring DCV is considered to be highly meaningful. Although BRT is said to be sensitive to the effects of psychotropic drugs on driving,^[44]^ eszopiclone, an optical isomer of zopiclone, had no effect on next-day BRT in both healthy and patient groups.^[45]^ However, most of these studies have involved healthy participants, and thus, further studies on different patient populations will be required in the future.

DS systems, although comprehensive, cannot reproduce actual traffic situations fully. For this reason, there are limitations in using a DS system to evaluate all types of driving performance necessary for actual traffic conditions. However, even actual vehicle tests regarded as a gold standard cannot evaluate all types of driving performance. Therefore, to promote road safety, it is important to accumulate evidence, including secondary evaluation items, and provide opportunities for patients and medical staff to discuss automobile driving while on medication.

If the residual effects of zopiclone as a positive control drug could be confirmed, the results as evaluated by the present DS system would be considered scientifically valid, which would make it possible to evaluate the effects of drugs on driving performance accurately in Japanese people, thereby providing useful information to both doctors and patients.

## Acknowledgments

The authors would like to thank the following employees at the Department of Clinical Development and Development Planning at Taisho Pharmaceutical Co., Ltd., who made valuable contributions to the development of the study protocol: Yumiko Imadera, Hiroshi Sunaga, Kazuma Nishiwaki, Chiaki Naruse, Yuusuke Miyazaki, Sayaka Hasegawa, and Hiroki Ogo.

## Author contributions

NO developed the study concept with MI, KI, DK and NT; MI and KI wrote the first draft of the manuscript, and DK, NT, MA and NO made critical revisions to the manuscript; all authors read and approved the final manuscript to be submitted.

Mari Iwata orcid: 0000-0001-6351-3602.
